# How to approach Monteggia-like lesions in adults: A review

**DOI:** 10.1016/j.amsu.2018.09.027

**Published:** 2018-09-25

**Authors:** Filippo Calderazzi, Cristina Galavotti, Alessandro Nosenzo, Margherita Menozzi, Francesco Ceccarelli

**Affiliations:** Department of Surgery, Orthopedic Clinic, Parma University Hospital, 43100, Parma, Italy

**Keywords:** Monteggia like lesions, Surgical approach, Tips

## Abstract

Monteggia-like lesions encompass a wide spectrum of fractures of the forearm and elbow associated with dislocations, subluxations and ligamentous lesions.

Many attempts have been made to classify these injuries, not only to understand their pathology but also to develop optimal treatments.

Unfortunately, although some of these classifications are complete, they are either complex, not immediately usable, or not exhaustive. An orthopedic surgeon who aims to rapidly treat this kind of injury needs a visual classification, and knowledge of the best surgical approach.

Monteggia like lesions do not allow for mistakes during surgery, as even a minor error could be prove detrimental to performing and completing all surgical steps.

In this paper, based on our extensive experience in treating these rare lesions, we suggest a practical guide to the best surgical approach for various types of Monteggia like lesions.

Some technical tips and pitfalls are also described.

## Highlights

• Make an accurate plan before surgery.
• Monteggia-like lesions do not permit mistakes in the surgical approach.
• The key-point of the Monteggia-like lesions is the pattern of the coronoid fracture.
• It is best to stabilize the ulnar fracture first.

## 1 Introduction

Since the first description in 1814 by Giovanni Battista Monteggia of a fracture of the proximal ulna associated with anterior dislocation of the radial head [1], the eponym of Monteggia fracture recently has included various patterns of complex fracture-dislocation of the proximal ulna and radius that are not yet well defined. In these types of lesions, the association of coronoid, olecranon, and radial head injury is common, whereby most of the bone structures implicated in elbow stability are disrupted.

Several classifications have been proposed for this condition. In 1959, Bado [2], classified the injury into four types based on the anterior, posterior, or lateral/anterolateral direction of the radial head dislocation.

The fourth type is associated with a fracture of the radius and ulna at the same level proximally and with an anterior dislocation of the radial head. More recently, however, Jupiter has further characterized and classified these complex injuries [3].

When these injuries are associated with radial head or coronoid fractures or complex patterns, they are named Monteggia variant, Monteggia like, or Monteggia equivalent [4,5].

Another classification called the Proximal Ulnar and Radial fracture-dislocation Comprehensive Classification System (PURCCS) was proposed by Giannicola et al., in 2013 [6]. This classification is based on the identification of six essential lesions, defined as main lesions. They must be recognized, since each of them affects the prognosis and requires specific treatment. The main lesions are as follows:

(1) Ulnar fracture, (2) Radio-humeral dislocation, (3) Ulno-humeral dislocation, (4) Proximal radio-ulnar dislocation, (5) Radial fracture and (6) Distal radio-ulnar joint/inter-osseus membrane lesion. The various possible combinations of these critical lesions explains the complexity and variability of their treatment.

All these classifications are helpful in planning the treatment of Monteggia-like fractures in terms of the type of fixation of different patterns of ulnar fractures, management of the radial head and ligamentous injuries, and suggesting the sequence of surgical steps. Accurate planning can allow the surgeon to achieve a stable fixation and early mobilization of the elbow, which is linked to good short- and long-term clinical outcomes.

Several surgical teams worldwide have published their experience; most of them provide suggestions and technical notes regarding the sequence of surgical steps and also data regarding possible early and late complications of these kind of lesions [4,[7], [8], [9], [10], [11]].

However none of these articles except one [10] emphasizes what should be the best surgical approach in the different types of Monteggia like lesions, maybe because there are many variables to consider.

Nevertheless, a misjudged surgical approach can lead to incomplete treatment; unstable fixation; prolonged surgical times; and excessive damage to soft tissues, nerves, or blood vessels.

In our department, within the last two years, we have treated 18 Monteggia-like lesions. We wish to achieve a minimum and meaningful follow-up before reporting the clinical and radiological outcomes.

However, based on our past mistakes, we have learned of several indications and techniques that concern various surgical approaches directly in the operating room.

Considering the recommendations of the specialized literature on this topic and of the principles of osteosynthesis, we present here some tips and tricks to an orthopedic surgeon not specialized in the elbow and dealing with this kind of injury.

### 1.1 Planning

Currently, an accurate imaging study of the lesion is mandatory. The radiographs performed after the trauma should not only include to the elbow, but also include the entire forearm and wrist. Radiographs performed after reduction of a possible elbow dislocation should also be studied. Finally, a CT-scan with 3D reconstruction of the injured region is required.

As Monteggia-like lesions are very difficult to analyze and understand, any single information obtained from different types of imaging modalities could be crucial.

1) Ulnar fracture: extension, comminution, articular involvement, type of coronoid fracture, if present, (simple or comminuted) and size of the main fragment, involvement of the sublime tubercle, involvement of the ulnar shaft.
2) Direction of the radio-humeral dislocation.
3) Presence of ulno-humeral dislocation or proximal radio-ulnar dislocation (extremely rare)
4) Radial head fracture: extension, comminution, size and position of the main fragments, involvement of the radial neck and/or radial shaft.
5) Distal radio-ulnar joint/interosseus membrane lesion.
6) Quality of the bone.
7) Cutaneous conditions and evaluation of soft tissue swelling.

All the above-mentioned questions should be addressed for accurate preoperative planning.

### 1.2 Patient preparation

All patients should be positioned supine, with the injured arm across the chest and with a high arm tourniquet. This allows the surgeon to develop the posterior universal extensile approach and the Taylor-Scham approach. A table should always be available so that it is possible to lay the forearm which allows the surgeon to develop Kocher's lateral approach or the Hotchkiss medial approach and to work more comfortably. Positioning the patients in a prone or lateral decubitus position is therefore unsuitable.

### 1.3 Surgical approaches

There are many described approaches for the proximal forearm and elbow, depending on the type of lesion [[12], [13], [14]].

The extensile posterior approach allows treatment of most of the distal humeral fractures and most of the simple or complex ulnar fractures, with any articular and shaft involvement [[15], [16], [17]].

The medial approach allows treatment of more specific ulnar fracture patterns, such as any type of coronoid fracture. In addition, it allows treatment of medial collateral ligament injuries. Many variants of the medial elbow approach are described, such as Hotchkiss, FCU (flexor carpi ulnaris) split, or Taylor-Scham [18].

A more anterior exposure is gained using the Hotchkiss Over-the-Top technique, but it is frequently used to address small coronoid fractures that remain anterior to the sublime tubercle [19]. This approach involves detaching a portion of the flexor pronator origin off the medial epicondyle.

**Fig. 1:**
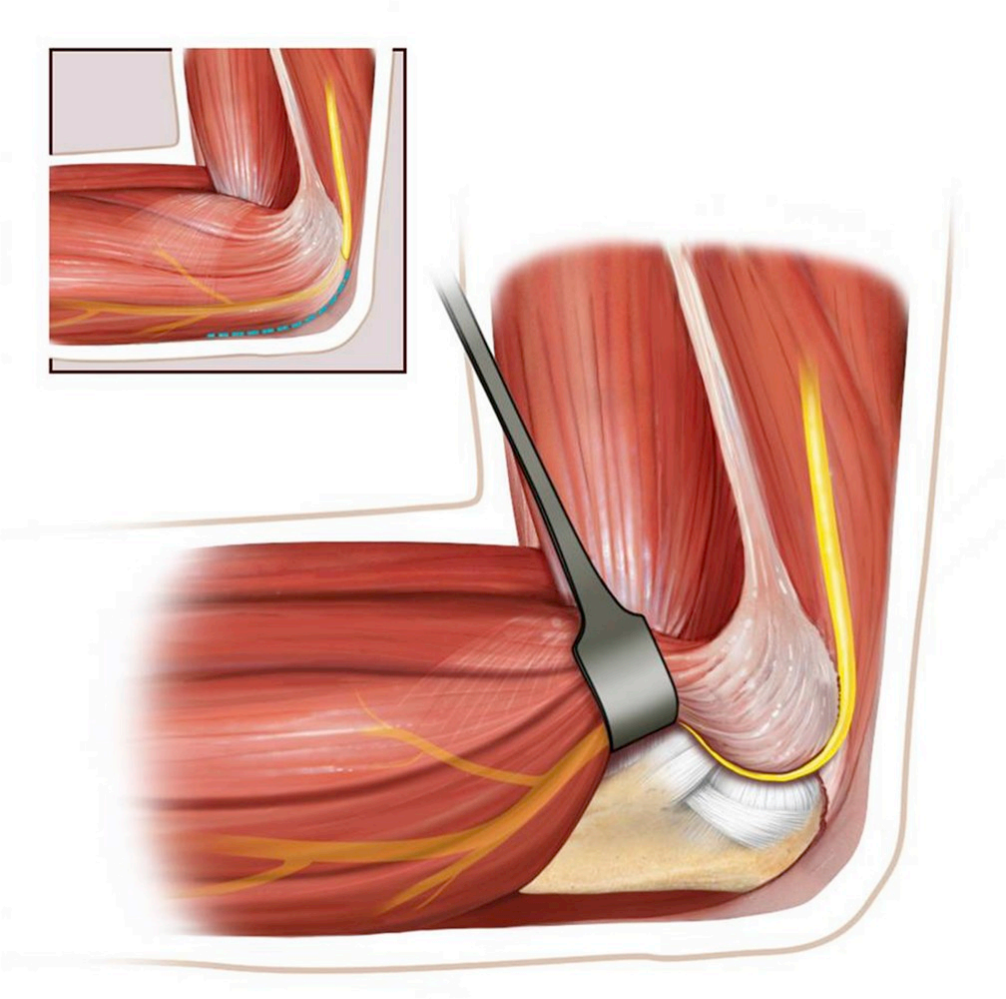
Draft of the Taylor-Sham approach (Courtesy of Istituti Ortopedici Rizzoli - Bologna, Italy).

Taylor and Scham [20] described elevation of the entire flexor-pronator mass off the medial ulna from a postero-medial approach, thus allowing the entire exposition of the coronoid process and of the proximal ulna (Fig. 1).

These approaches can be chosen based on the fracture pattern of the coronoid.

The lateral approach allows treatment of radial head and neck fractures of any type. In addition, it allows treatment of lateral complex ligament injuries.

Many variants of lateral elbow approaches have been described, such as Kocher, Kaplan or Midaxial [12,13].

The Kocher approach uses the interval between the anconeus and the extensor carpi ulnaris (ECU). It can be extended proximally and is safe because the ECU protects the radial nerve. However, in this approach care must be taken to identify and preserve or if necessary, repair the lateral ligamentous complex [21].

A surgeon who deals with Monteggia-like lesions should know the posterior, Hotchkiss, Taylor-Scham, and Kocher approaches.

### 1.4 Choice of surgical approaches

The above-mentioned approaches are usually performed to treat a bone fracture -simple or complex- or a ligament disruption. They allow to treat no more than two different simultaneous lesions. Unfortunately, in Monteggia-like lesions the number of the injuries (three or more) is such that it is difficult to treat them all using a single surgical approach as originally described.

**Fig. 2:**
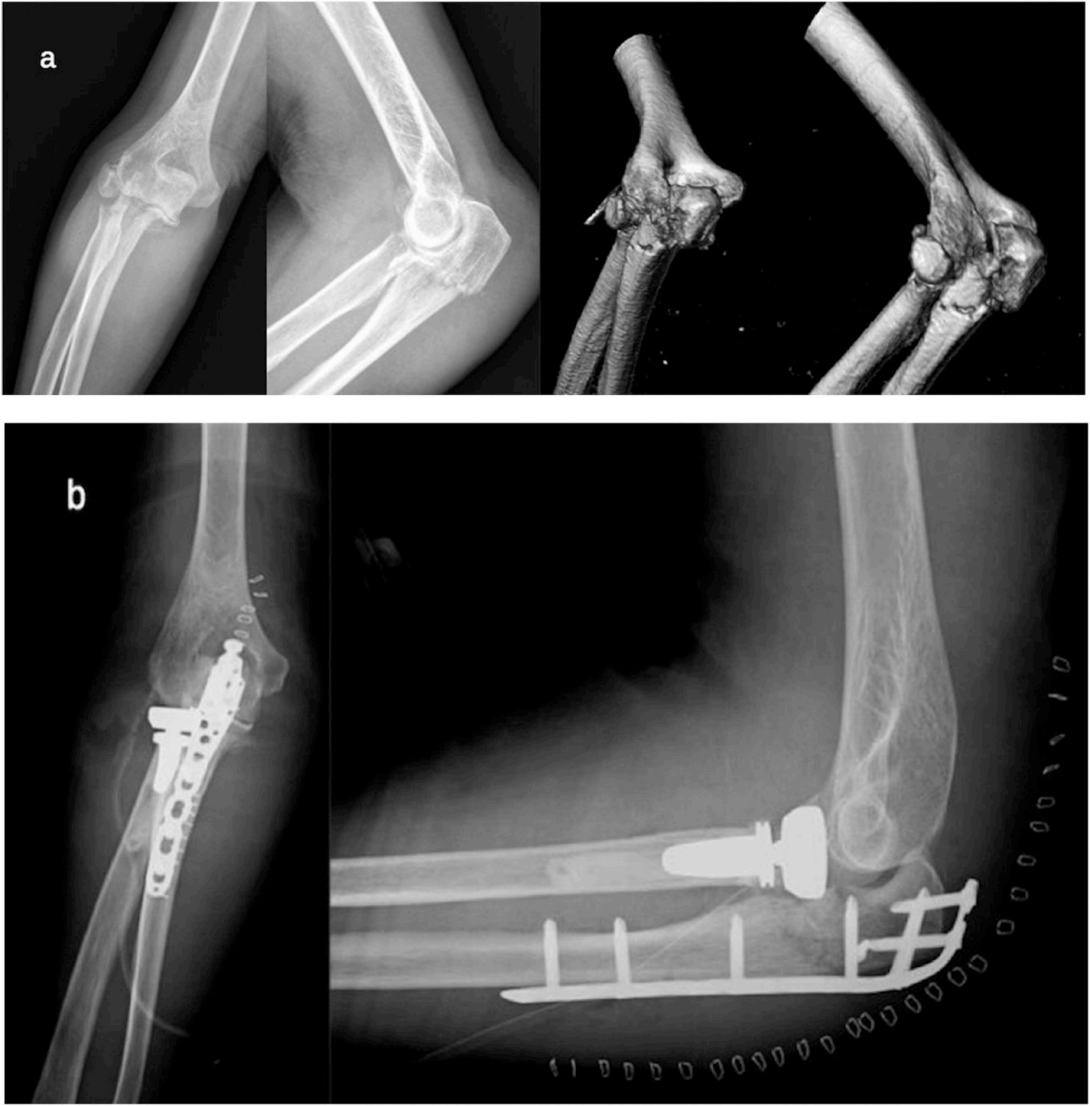
a) Pre-operative radiography and CT scan: simple fracture of the proximal ulna and comminuted fracture of the radial head in osteoporotic bone in a 74- year-old woman. b) Post-operative radiography: global posterior approach and subcutaneous Kocher approach– Pre-contoured locking plate and cemented radial head prosthesis.
- *Simple and complex proximal olecranon and/or metadiaphyseal ulnar fracture + fracture – subluxation of radial head + coronoid fracture (large fragment) (*Fig. 3a).

Here, we provide some practical tips based on the most frequent patterns of Monteggia-like lesions:

- *Simple and complex proximal olecranon and/or metadiaphyseal ulnar fracture + fracture – subluxation of radial head – No coronoid fracture* (Fig. 2a).

**Fig. 3:**
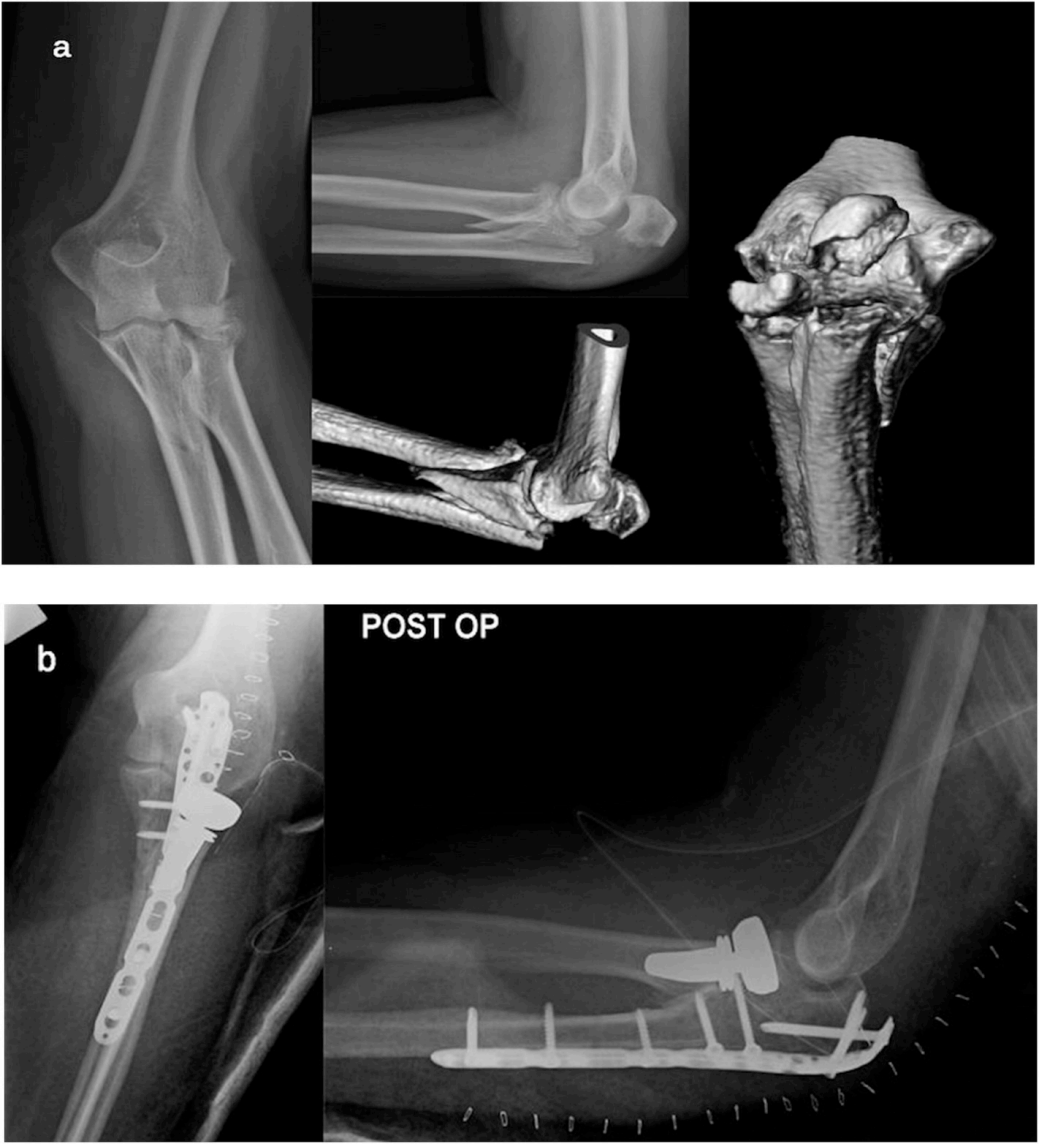
a) Pre-operative radiography and CT scan: fracture of the proximal ulna and coronoid fracture (large fragment); comminuted fracture of radial head in a 66-year old woman. b) Post-operative radiography: global posterior approach and subcutaneous Kocher approach – Free lag screws for coronoid fracture. Pre-contoured locking compression plate and radial head prosthesis.

In these patterns, there are two possible approaches:

The first one is the extensile posterior approach [13]. It is a “global approach” that enables the management of complex reconstructive and traumatic conditions of the elbow, especially fracture-dislocations. The surgeon can obtain circumferential exposure of the elbow, including the collateral ligament complexes, anterior joint capsule, and coronoid process through a single skin incision [22], using various intermuscular approaches as previously described. It is versatile and flexible and does not endanger the stability of the joint or the surrounding neurovascular structures. This single skin incision precludes any other incision around the elbow, in order to avoid the risk of skin necrosis [23].

This approach allows the performance of any type of osteosynthesis (posterior pre-contoured plate, tension banding cerclage) of any pattern of ulnar fracture. It is also possible to reduce and stabilize a large coronoid fragment using screws, pins, or a small fragments plate (Fig. 3b and c).

Tips: In the case of extreme comminution or severe osteoporosis, it is useless to attempt an anatomical reduction. Usually, the ulnar fracture is the first to be treated; if this step is too long there is the risk that the overall surgery time will be too long. It is better to proceed with a bridging plate: it is essential to restore the rotational and longitudinal alignment, and ulnar length.

**Fig. 4:**
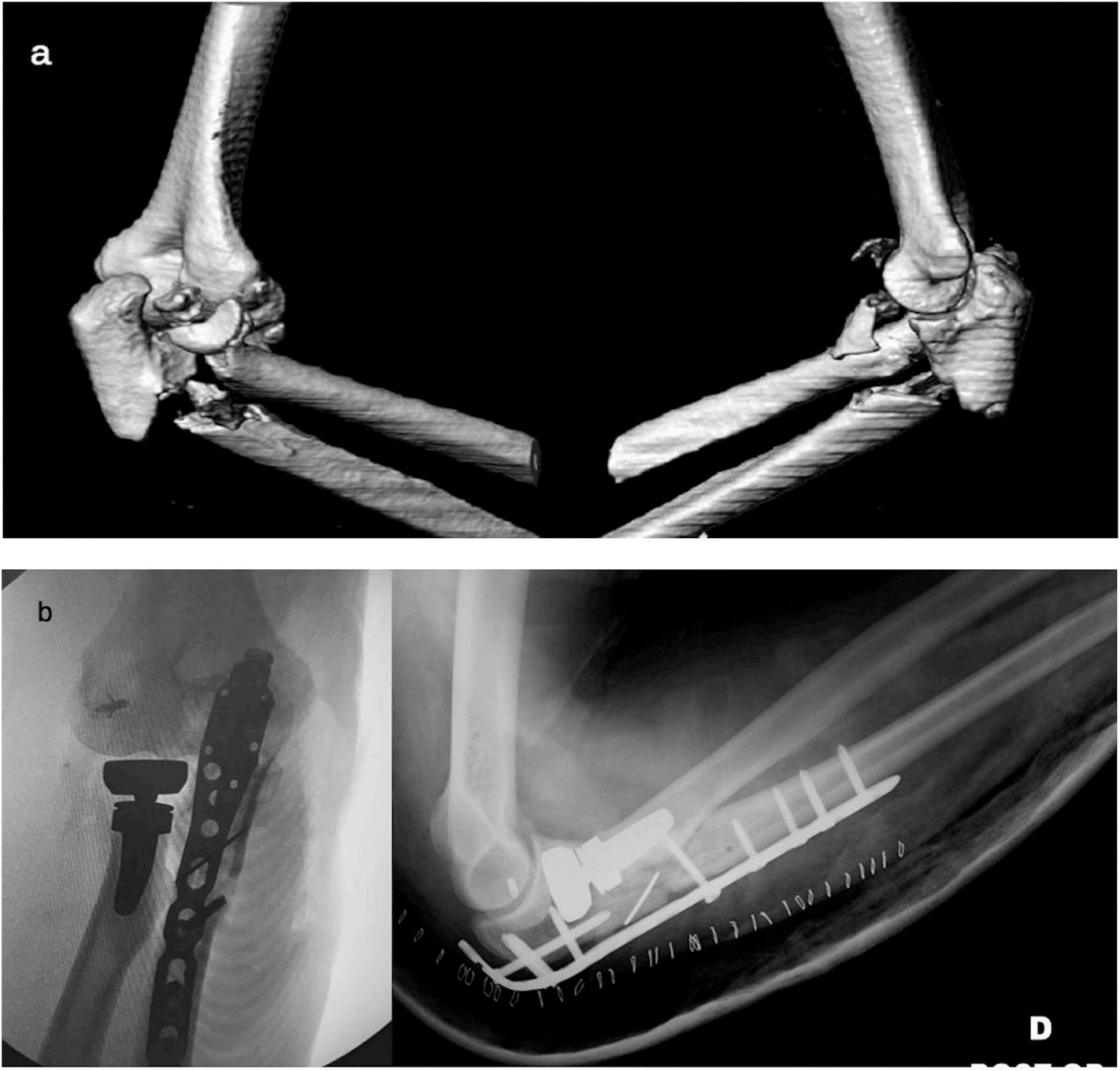
a) 3D-CT scan: comminuted fracture of the proximal ulna and of the radial head in a 58-year old man. b) Post-operative radiography: global posterior approach and subcutaneous Kocher approach – Pre-contoured locking compression plate, cemented radial head prosthesis and suture anchor fixation of lateral ulnar collateral ligament.

Subsequently radial head fracture-dislocation is treated. Taking advantage of the posterior universal approach, it is possible to develop the tendineous interval of Kocher (through the same skin wound) and to manage the radial neck and/or head fracture using osteosynthesis or a prosthesis, (less frequently, excision) (Fig. 2-b; Fig. 3-b). It is also possible to treat a lesion of the ligamentous lateral complex (such as the anular ligament and/or the lateral ulnar collateral ligament) (Fig. 4a–b).

We do not completely agree with Matar et al. [10]. They stated that radial head fractures can be reached through the posterior extensile approach, taking advantage of proximally reflecting the concomitant olecranon fragment (if it is present) by using the attached triceps tendon and flexing the forearm. This should allow good visualization of the radial head, thus enabling anatomical reduction or replacement. We believe that it is not always possible to reflect the olecranon fragment, as this depends on the type and comminution of the fracture. In addition, assuming it is possible to reflect the olecranon fragment, performing an osteosynthesis of the radial head is technically very demanding, because of the very narrow spaces.

The development of Kocher's interval, if carried out starting from an extensile posterior cutaneous approach, is not always easy to perform. In particular, if an osteosynthesis has been decided upon, enough space is needed to operate. Therefore, we advise that surgeons not hesitate to completely detach and raise the anconeus to locate all radial head fragments that are often intra-articular and far from the original position. We must point out that the development of Kocher's interval from the posterior cutaneous approach necessarily involves a large dissection of the subcutaneous tissues.

**Fig. 5:**
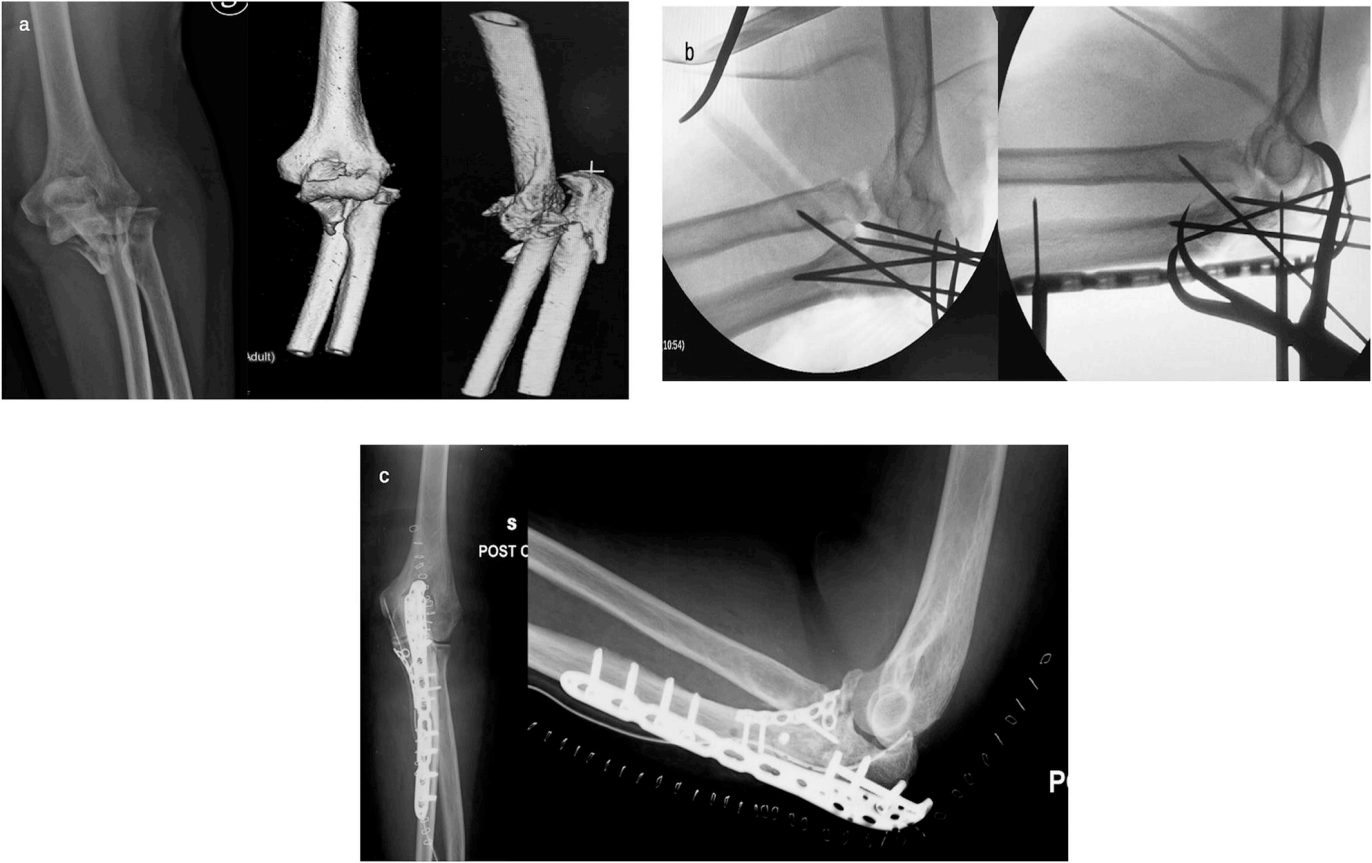
a) Radiographs and 3D-CT scan: fracture-dislocation of ulno-humeral joint, comminuted fracture of proximal ulna and sigmoid notch; large fragment of coronoid process. Fracture of the radial head with a small fragment. Osteoporosis. The patient was an 86-year-old woman. b) Intraoperative fluoroscopy: Taylor-Scham approach - temporary fixation of the fragments with clamps and K-wires. c) Post-operative radiography: Pre-contoured locking compression plate and 2.4 T buttress plate for coronoid fracture. Removal of the small fragment of the radial head.

**Fig. 6:**
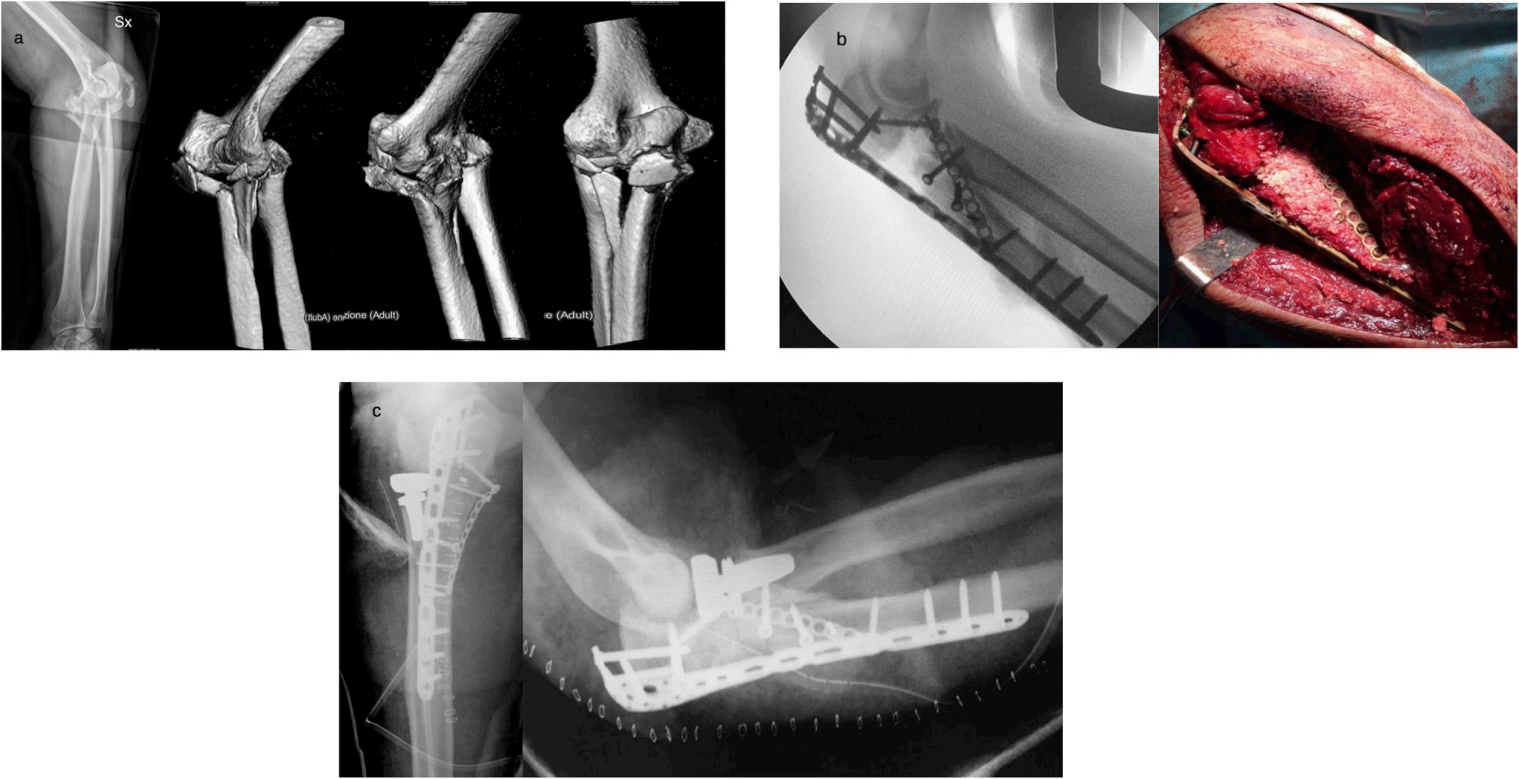
a) 3D-CT scan: high comminution of proximal ulna, sigmoid notch; large fragment of coronoid process. Comminuted fracture of the radial head. The patient was a 58-year-old woman. b) Intraoperative fluoroscopy and surgical field: The Taylor-Sham approach and standard Kocher approach - filling of cancellous gap with autograft obtained from the excision of radial head fragments and morselized homologous bone. c) Post-operative radiograph: Pre-contoured locking compression plate, free screws, headless 18-mm screw and 2.0 buttress plate for coronoid fracture; Radial head prosthesis.

The second possibility encompasses the development of two different cutaneous surgical approaches. The first is the Taylor-Scham approach [20]: it allows good exposure of the entire proximal ulna, thereby allowing a surgeon to fix the olecranon, coronoid, sigmoid notch and metadiaphyseal fragments. In the presence of sigmoid notch fragmentation, the use of bone graft and/or bone growth enhancers [24,25] might be considered to sustain the articular surface and obtain a stable internal fixation (Fig. 5a–c; Fig. 6a–c).

In this case it is possible to carry -out a standard Kocher approach [21] to treat radial/neck fracture-dislocations, because it provides a good exposure of the proximal radius (Fig. 6 c).

These two cutaneous approaches involve incisions that are located a suitable distance from each other so that they do not pose a risk of causing skin necrosis [26]. We suggest that a surgeon uses the double approach if he is not sufficiently skilled at performing the universal posterior approach and if he desires to have a good view of the entire lateral side of the proximal forearm. This also enables the location and removal of all intra-articular radial head and neck fragments.

**Fig. 7:**
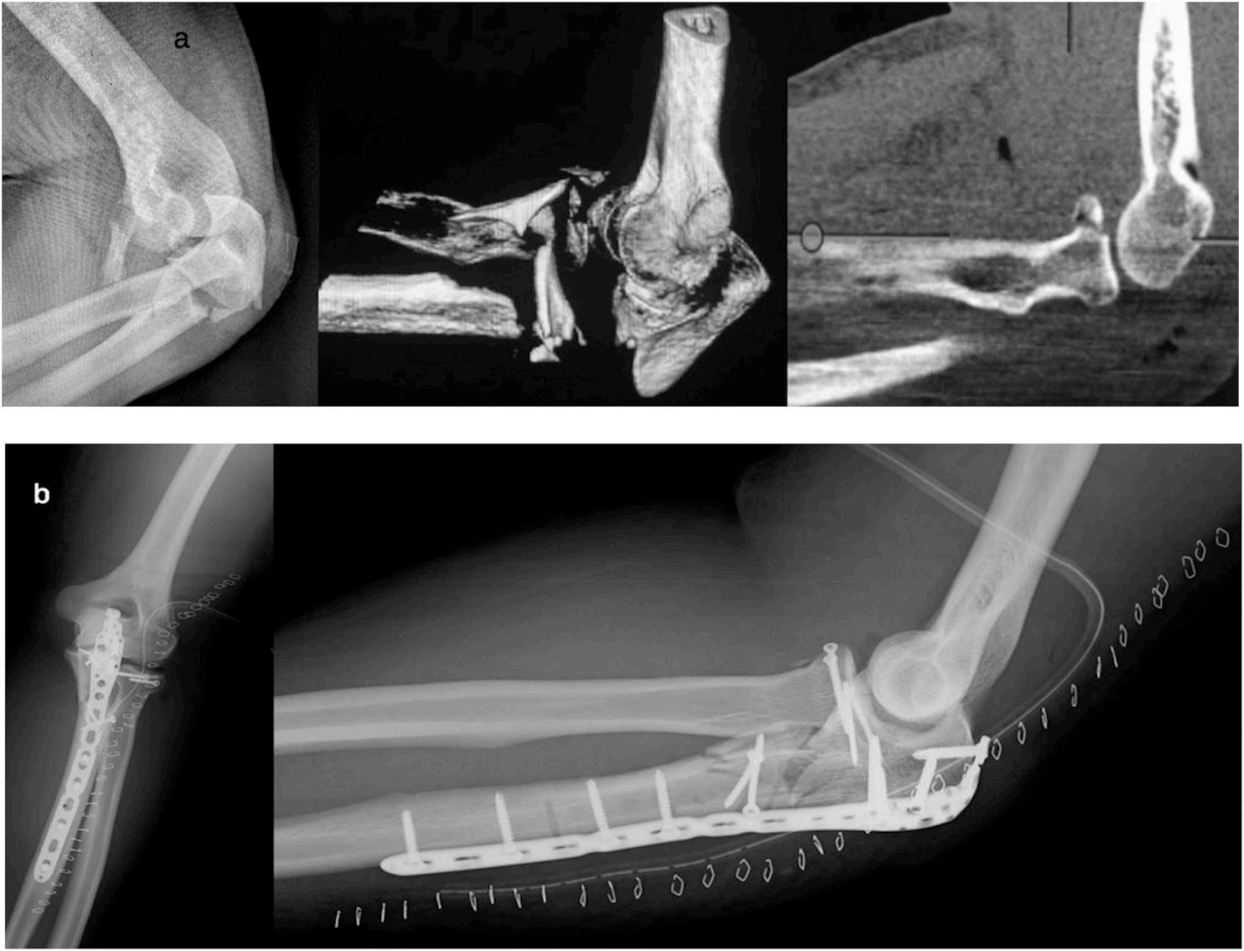
a) Radiographs and CT scan: comminuted fracture of the proximal ulna and coronoid process. Simple fracture of the radial head in a 26-year-old man. b) Radiographs at 9 months after surgery: global posterior approach and subcutaneous Kocher approach – Pre-contoured locking compression plate, two lag 2.7 mm screws and two loose K. wires for synthesis of comminuted coronoid process. Synthesis of the radial head with 2 small fragments screws (2.4 mm). Fracture healing; presence of heterotopic ossifications.

*Simple and complex proximal olecranon and/or metadiaphyseal ulnar fracture + fracture – subluxation of radial head + coronoid fracture (comminuted)* (Fig. 7a).

In this pattern, the “universal” posterior extensile approach is preferred, as it allows for the fixation of various ulnar fracture patterns. In this pattern, the coronary process is fragmented and/or the main fragment is not so large and therefore, it is very difficult to reach and synthesize it from the back. It is hence necessary to develop Hotchkiss' medial approach after the osteosynthesis of the ulna (through the same skin wound), to provide good visualization of the whole coronoid process and its stable fixation (screws, pins, or suture anchors).

We do not entirely agree with Matar et al. [10] who stated that all types of coronoid fracture can be reached using the posterior extensile approach, taking advantage of proximally reflecting the concomitant olecranon fragment by using the attached triceps tendon. This should allow excellent visualization of the coronoid fracture, thus enabling anatomical reduction.

As stated earlier, we believe that it is not always possible to reflect the olecranon fragment, as this depends on the type and the comminution of the fracture. In addition, even if it is possible to reflect the olecranon fragment, it is not always possible to synthesize the coronoid process with ulnar proximal metaphysis, even just temporarily, given that this also depends on the pattern and comminution of the coronoid fracture.

Consequently radial head fracture-dislocation is treated as previously described.

Simple and complex proximal olecranon and/or metadiaphyseal ulnar fracture + eventual coronoid fracture (any type) + fracture – dislocation of the radial head + fracture of the radial shaft.

Taylor-Scham's postero-medial approach is the best approach for ulnar fractures. To treat radial head fracture-dislocation and concomitant radial shaft fracture we recommend Thompson's dorso-lateral approach [27] as it enables the treatment of both injuries.

### 1.5 Hardware

**Fig. 8:**
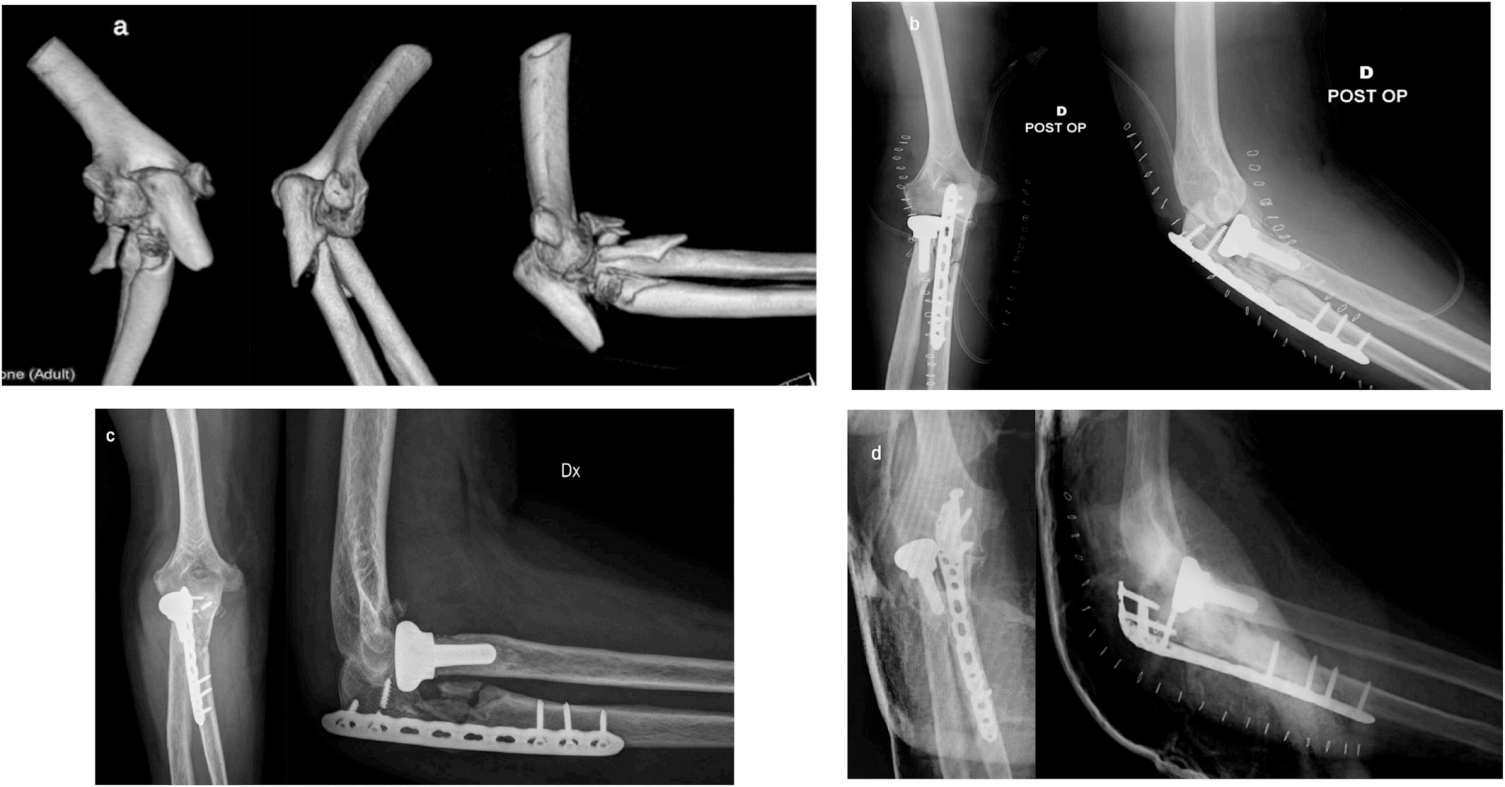
a) 3D-CT scan: comminution of the proximal ulna, coronoid and of the radial head in osteoporotic bone in a 72-years old woman. b) Post-operative radiographs: global posterior approach - initial attempt to perform anatomical synthesis of comminuted osteoporotic fragments with consequent biological damage – Weak final bridging plate fixation (No locked screws). - cemented radial head prosthesis. c) Radiographs at 3 months after surgery: Breakage of one screw, implant loosening. d) Post-operative radiographs after revision surgery: Pre-contoured bridging plate, iliac crest graft.

A proper pre-operative planning should also consider the type of synthesis to be performed. For the proximal ulnar fractures involving the olecranon process and the sigmoid notch, most often, it is necessary to apply the posterior pre-contoured locked angle plate [28,29]. In the elderly with osteoporosis and in patients with high comminution of fracture, this device should be applied as a bridging plate, avoiding synthesizing all single fragments and so causing a biological damage to the vascular supply. An inappropriate fixation could lead to implant failure (Fig. 8a–d).

In younger patients with a simple fracture patterns or with large fragments, anatomical reconstruction is recommended [28,30], by the use of small fragment free lag screws measuring 2.4–3.5 mm, and a proximally contoured 3.5 mm. Locking compression plate should be applied as a protection/neutralization device [30]. This construct is made in order to achieve a stable fixation and to allow early recovery [11]. An early temporary fixation is provided by the use of clamps and k-wires [31] (Fig. 5b).

If a coronoid process comminuted fracture is present, it is suitable to synthesize it by the use of small fragment screws measuring 2.0–2.7 mm, suture fixation, suture anchors, or **small** fragment reconstruction plates or T-shaped miniplates measuring 2.4–2.7 mm [29]. That are more moldable and play a role as buttress devices (Fig. 5c).

Radial head fractures should be addressed after temporary or definitive restoration of ulnar length and alignment/rotation. This allows the radial head to be appropriately sized [11].

If the synthesis is possible, small fragments screws (1.5–2.4 mm) or headless screws (1.5–3.0 mm) [[32], [33], [34]] should be used (Fig. 7b); if there is an involvement of the radial neck, a small fragment hand plate or a pre-contoured locking low-profile plate are appropriate (2.0–2.4 mm) [35]. However, in cases of high comminution, a radial head prosthesis is highly recommended [[36], [37], [38], [39], [40], [41], [42]]. Anyway, it is advisable to start this surgery with radial head prosthesis in the surgery room. There is no scientific evidence of superiority in terms of effectiveness or survival of bipolar or monoblock radial head prosthesis as well as the use of cemented or pressfit implants [[37], [38], [39], [40], [41], [42]]. Radial head excision should be avoided, as the radial head is a primary stabilizer of the elbow and forearm, unless the patient is not very functionally demanding or the amount of fragmented radial head to be removed is small [34,43].

Finally, if a residual soft tissue laxity is still present, a ligamentous suture of medial and/or lateral elbow compartment is recommended, with direct stitching or suture anchors [11] (Fig. 4b).

Persistent instability or irreducibility of the radial head may also be related to an ulnar shortening; in these cases it is essential to assess the ulnar alignment.

The Essex-Lopresti lesion is frequently associated with Monteggia-like lesions; in this condition, the distal radio-ulnar joint (DRUJ) stability must be also evaluated. If instability of DRUJ persists after treatment of the radial head, DRUJ pinning and/or the repair of triangular fibrocartilage complex (TFCC) may be indicated [[44], [45], [46]].

After osteosynthesis and ligamental reconstruction, the elbow should be evaluated under fluoroscopy to verify the recovery of ulno-humeral, radio-humeral and proximal radio-ulnar joint stability. The dynamic elbow fixator should be considered in cases of persistent instability of the ulno-humeral joint or unstable osteosynthesis of small articular fragments [43].

### 1.6 Post-operative care

In the post-operative period, cryotherapy, antideclive position and good analgesia are essential to allow an early motion of the joint. For an early rehabilitation a stable reconstruction of the fractures is also essential [43].

In all the cases in which the fixation has been considered stable by the surgeon, passive and active mobilization should be started within the first 3 days.

Mobilization should be allowed both for flexion/extension and for supination/pronation of the elbow, as tolerated based on the pain. It should be borne in mind that extreme grades of motion should be avoided for the first two weeks to avoid excessive stress to the surgical wound.

In other cases, in which an incomplete stable fixation has been reached or some ligamentous reconstruction has been performed or in presence of osteoporotic bone, the elbow should be rested in a back-slab at 90° for at least 2 weeks. At the end of this period, radiographs are obtained and if stability is radiographically confirmed, these patients should start a progressive range of motion exercises, both in flexion/extension and supination/pronation. Those with associated comminuted coronoid fractures should be mobilized in a hinged brace for the first three weeks [8]. Physiotherapy should be continued for at least 6 weeks.

Strengthening exercises and progressive full weight bearing should be allowed 8–12 weeks postoperatively, based onradiographic evidence and patient compliance [9,10].

In literature, the optimal strategy for prevention of post-traumatic heterotopic ossifications is still a topic of debate. However, following the current major evidences [47], in order to minimize ectopic bone formation, non-steroid anti-inflammatories (indomethacin 25 mg; orally 3 times daily) should be administered at least for 3–4 weeks post-operatively [9,43].

### 1.7 Tips

- Make an accurate plan before surgery.
- All the necessary hardware and tools should be available.
- In the posterior approach, medial and lateral full thickness skin flaps should be raised in order to allow adequate fracture visualization and reduce the risk of wound necrosis.
- If there is an ulno-humeral dislocation/instability, do not hesitate to apply a K. wire for temporary arthrodesis: this allows to expose and locate various anatomical structures more safely and ensure better anatomical relationships.
- The key-point concerning Monteggia-like lesions is the pattern of the coronoid fracture.
- It is best to stabilize the ulnar fracture first.
- The osteosynthesis of the ulna should be excellent in terms of stability and length/rotation restoration. In most cases this allows a reduction of radio-humeral instability and offers a good landmark for a proper size of eventual radial head prosthesis.

## Ethical approval

Nothing to declare.

## Source of funding

This study has not been financially supported.

## Author contribution

**Filippo Calderazzi**: Study design, writing.

**Cristina Galavotti**: Study design.

**Alessandro Nosenzo**: Data collection.

**Francesco Ceccarelli**: Study design, supervision.

**Margherita Menozzi**: Data collection, writing.

All authors contributed to and approved the final version of manuscript.

## Conflicts of interest

All authors declare there are no conflicts of interest.

## Research registration number

None.

## Guarantor

Filippo Calderazzi.

## Provenance and peer review

Not commissioned externally peer reviewed.

## Consent

Nothing to declare.
